# Ligament pre-tension determines outcome in sacroiliac joint in silicon modelling

**DOI:** 10.1007/s11517-025-03396-w

**Published:** 2025-06-17

**Authors:** Mark Heyland, Hendrik Schmidt, Friederike Schömig, Daven Maikath, Dominik Deppe, Matthias Pumberger, Georg N. Duda, Katharina Ziegeler, Philipp Damm

**Affiliations:** 1 Julius Wolff Institute, Berlin Institute of Health at Charité – Universitätsmedizin Berlin, Berlin, Germany; 2 Centrum Für Muskuloskeletale Chirurgie (CMSC), Charité – Universitätsmedizin Berlin, Berlin, Germany; 3 Department of Radiology, Charité – Universitätsmedizin Berlin, Campus Mitte, Berlin, Germany; 4 Department of Radiology and Biomedical Imaging, University of California San Francisco (UCSF), San Francisco, USA

**Keywords:** Spine, Sacrum, Biomechanics, Ligament elasticity, Finite element model sensitivity

## Abstract

Biomechanical analyses of the sacroiliac joint (SIJ) are limited. We hypothesize that influence of ligament pre-tension on strain and relative joint movement is morphologically sex-specific and more pronounced than effects of body weight. Finite element models were developed from CTs of a larger cohort (*N* = 818) for typical male (TMJ) and typical female joint (TFJ) geometries. For different loading scenarios, stresses were higher in TFJ than TMJ for same pre-tension, only considering sex-specific morphology. Loading in antero-posterior direction caused highest stresses and relative movement. Ligament pre-tension was most sensitive with mean sensitivity factor (change output [%]/change input [%]): 71.04/33.64 for translation, 43.09/4.02 for rotation, 2.11/ − 8.97 for stress for TFJ/TMJ respectively. Mean sensitivity factor of ligament stiffness was − 1.14/ − 1.06 for translation, − 0.90/ − 0.89 for rotation and 0.17/0.13 for stress, while mean sensitivity of load intensity was 1.09/1.10 for translation, 0.91/0.88 for rotation and 0.54/0.58 for stress for TFJ/TMJ respectively. Relative motion was more sensitive to parameter variations than stress. The hypothesis was confirmed: influence of ligament pre-tension on stress but especially relative joint movement of SIJ is morphologically sex-specific and larger than body weight effects. As this may play a crucial role in pain development, ligament pre-tension must be verified in situ in the future.

**Graphical abstract:**

Sacro-iliac joint models are determined by ligament laxity: stiffness & pretension. Our analyses show the relative effect of parameter assumptions on modeling results. A substantial preload or pretension changes joint stability / mobility and stress. The influence of ligament pre-tension on stress and relative joint mobility of the sacro-iliac joint was larger than load intensity (bodyweight of patient) in our modelling set-up. Evaluation of the specific ligament pretension is imperative for patient-specific finite element models.
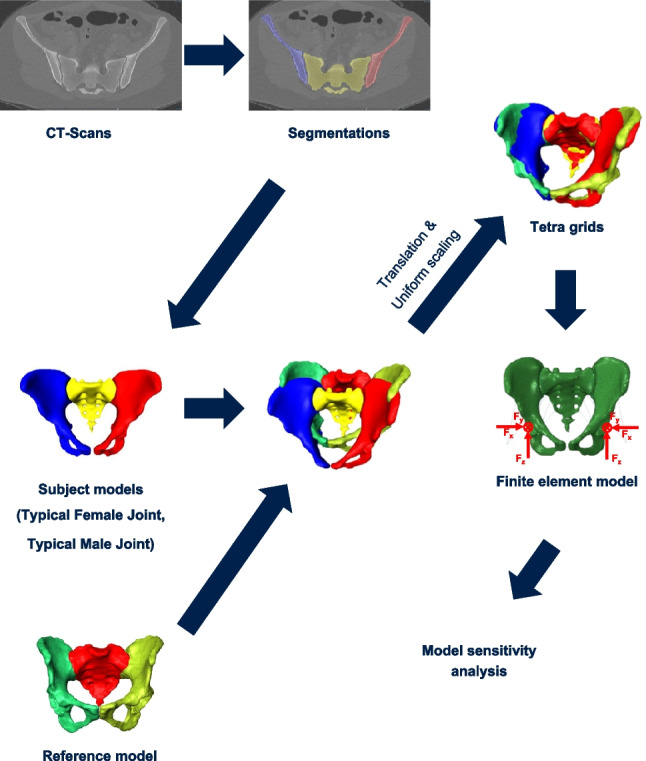

## Introduction

Low back pain (LBP) is a major health care challenge as up to 80% of people experience LBP at least once during their lifetime. Even though the sacroiliac joint (SIJ) is reported to be the cause of up to 30% of LBP cases [3, 26], and although it is assumed that a dominant cause is mechanical in nature, biomechanical analyses are rare. Anatomically, the SIJ constitutes a major articulation that enables force transduction from the pelvis and lower extremities to the spine or reversed [28].

Mechanical disease of the SIJs may have different causes, but one possible explanation for a degenerative effect is a change in passive or active structures (e.g. joint morphology, ligaments and muscles) leading to variations in load magnitude and load direction, and thus locally increased joint movement or increased mechanical stresses. This may lead to chronic LBP with high patient burden. When ligamentous laxity is prevalent, i.e. ligaments are elastic due to low stiffness and do not exhibit significant pre-loads, especially the interosseous and posterior ligaments, then relative joint movement may occur under smaller loads, especially shear movements. However, also under larger loads, tissue strain could be altered. As the SIJ is stabilized by different muscles and ligaments [10, 11], joint motion is restricted but has been found to be increased in patients with degenerative spinal disorders [20, 21]. Contact zones in the SIJ that are most affected by degenerative changes have been postulated to undergo altered mechanical straining. Furthermore, it is assumed that ligament pre-tension changes joint loading. A study with fresh samples by Steinke et al. [24] assessed the properties of the sacrotuberous ligament in cadaver specimens and found substantial pre-load in the sacrotuberous ligament in situ. Ligament pre-load was so far not considered in most in silicon studies and currently does not represent a well-known factor of influence in computer models of the SIJ or lower spine. The exact pre-tension in ligaments is still unknown possibly due to the difficulty in non-invasively measuring these in vivo forces and the historical focus on other joints and large muscle forces. The effects of differences in pre-tension are not well-documented. Potentially, ligament pre-tension in the SIJ is clinically significant for understanding pain mechanisms, differentiating SIJ pain and eventually tailoring treatments to address instability or excessive tension, although in vivo assessment is currently limited and requires further research to guide diagnosis and therapy. Recognizing sex-specific differences in the impact of pre-tension may lead to more personalized approaches. While it has been established that pre-tension of (spinal) ligaments influences the kinematics of the finite element (FE) model [9], it has been suggested before that there still is a lack of knowledge of appropriate pre-tension levels for the different (spinal) ligaments [4]. However, the effects of pre-tension especially at the SIJs remains uncertain [13, 24].

This work is a foundational study exploring the feasibility and potential significance of considering ligament pre-tension and sex-specific morphology in SIJ modeling. This study therefore aimed to develop a finite element model of the SIJ to (1) understand how different input parameters change joint loading and (2) investigate the effect of ligament pre-tension on joint surface stress and relative motion. It was hypothesized that the influence of ligament pre-tension on stress and relative joint movement of the SIJ is morphologically sex-specific and the effect of changes in ligament pre-tension is more pronounced than that of load intensity in terms of body weight.

## Methods

**Fig. 1:**
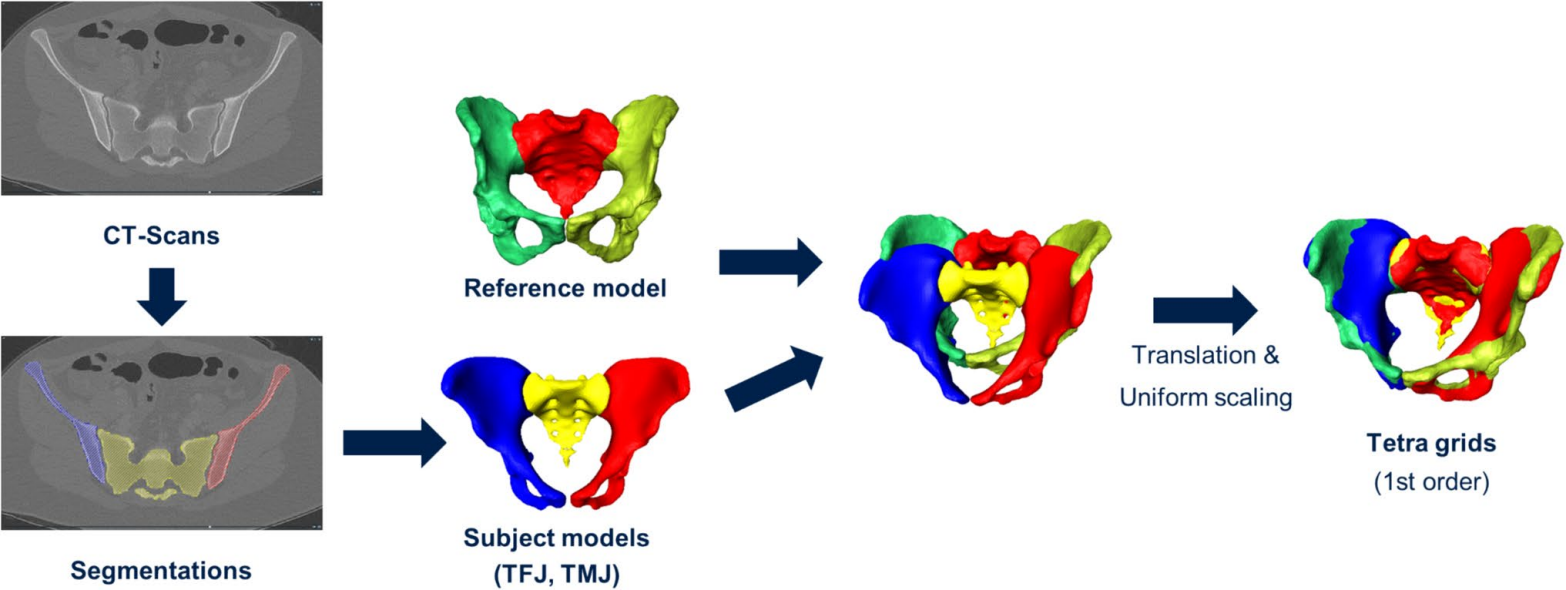
Model geometry (TFJ, TMJ) was derived through segmentation from CT scans. For consistency, all models were transformed into the reference model coordinate system and uniformly scaled to the reference model size

The retrospective evaluation of imaging data was part of a study that was approved by the local ethics committee (EA1/300/19). We retrospectively included 818 patients without known SIJ disease who had received computed tomography (CT) imaging of the pelvis with 0.5-mm sharp bone kernel reconstruction [31]. An experienced radiologist selected two typical joint morphologies based on their expert assessment of the most frequently observed morphological characteristics within the 818-patient cohort: one typical female joint (TFJ) and one typical male joint (TMJ). Both used geometries do not represent multiple individual patients but rather simulation variations using two representative geometries. With semi-automatic segmentation using the Amira Software (Zuse Institute Berlin & Thermo Fisher Scientific, 2021), the CT scans of the pre-selected patients were computed into usable geometric shapes that were also smoothened and meshed to 3D volumes of bone. Those tetrahedral grids were used for further FE analysis in Abaqus/CAE 2019 as FE pre-processor (Dassault Systèmes Simulia Corp., 2019). The grids of all CT scans were transformed into the same reference coordinate system, which was obtained from a reference model of a female patient with an instrumented hip implant [7] on the left side (H5L) (Fig. 1). No difference in loading between the sexes could be detected before relative to bodyweight, and therefore, the data from H5L were transferred to both sexes in order to standardize the influence of the load against the background of the joint morphology [1, 2, 6]. This reference model also provided the data for the loads that were applied to the models, as those joint loads were directly measured in vivo using an instrumented, telemetric implant (OrthoLoad, https://orthoload.com) [2, 8]. We specifically chose loads of only one specific patient and only one specific representative trial measurement during walking instead of averaged loads of multiple patients or multiple trials to ensure real physiological loading, as averaging may introduce unrealistic effects such as aliasing, which eliminates the individual loading patterns.

The rationale for selecting this specific patient was to utilize a realistic, in vivo measured loading scenario, avoiding potential issues associated with averaged or generic load profiles. This approach is a first step towards a musculoskeletal modeling system for specific male and female patients, but at this stage we wanted to investigate a first impression with a focus on ligament laxity. If averaging approaches are chosen, also for the joint geometries, it is important to also analyze the most distinct statistical variations, e.g. by using a principal component analysis, and not just analyze the averaged model. However, assigning associated loads to the individual geometries is not trivial and requires additional assumptions, for instance by using additional musculoskeletal models. There are a number of covariates and in a first step we are only interested in the relative biomechanical effects and associated sensitivities, but in a physiological set-up, we chose a different approach. We vary the covariate of morphological sex differences in two typical set-ups, which use scaled, distinct, but patient-specific geometries, and we uniformly scale those geometries to a known pelvis while preserving the typical SIJ morphology. Then, we apply the known specific, measured realistic loads that belong to same patient with the known pelvis geometry. This way, we ensure that the loads and geometries remain realistically paired, but we can test the relative effects of different joint morphologies and their biomechanical environment at the same time.

### Material properties

**Fig. 2:**
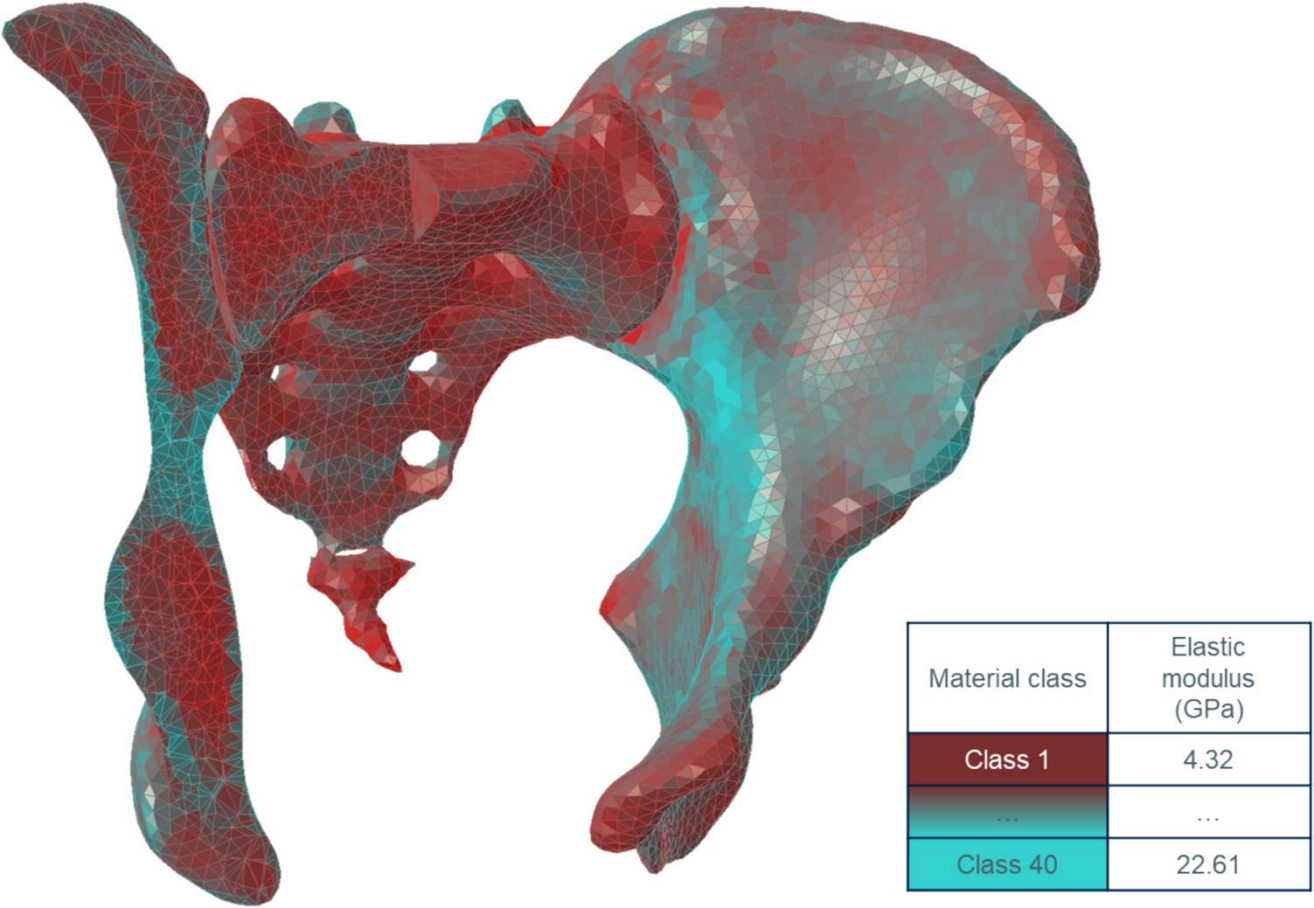
Quarter-cut view of the model (TFJ) with color mapping of the elastic modulus. Cyan-colored elements have higher elastic modulus; red-colored elements have lower elastic modulus. Elastic modulus values range from 4315 to 22608 MPa (40 material classes)

**Fig. 3:**
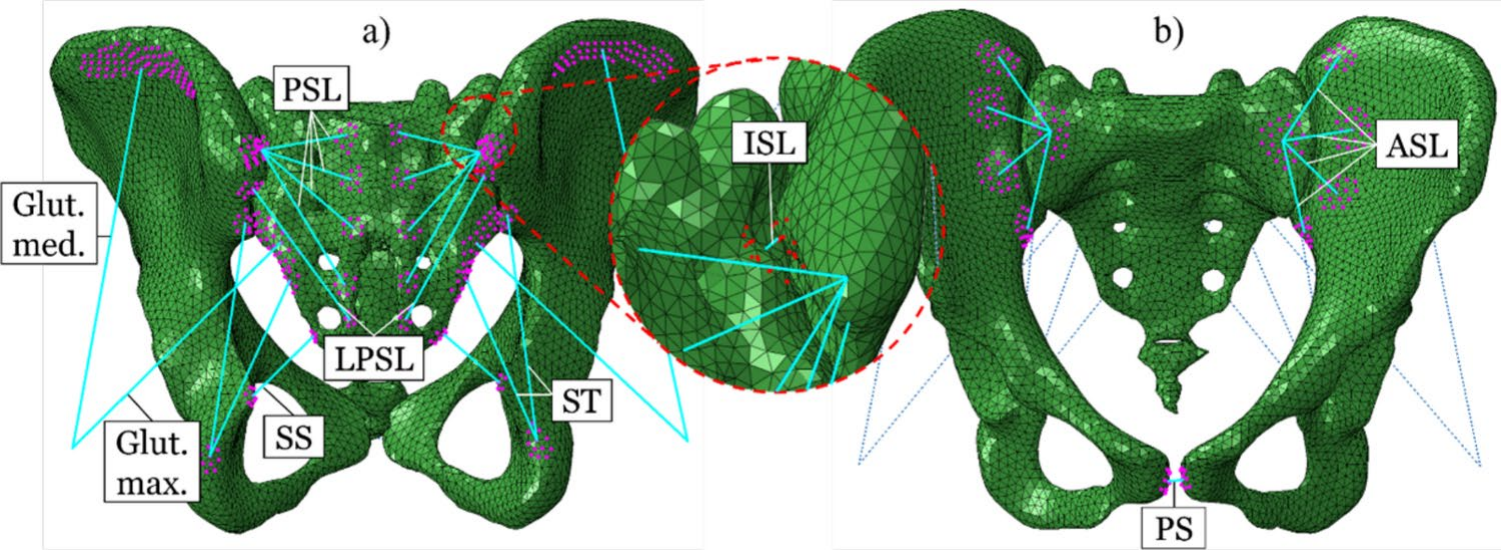
Tension-only and pre-loaded springs representing the ligaments and muscles (in cyan color, while specific attachment points were highlighted in purple, in detail view of the interosseous sacroiliac ligament attachment points are in red), also see Table 1. To include the main sources of sacroiliac joint stabilization, the sacrotuberous ligament (ST), the sacrospinous ligament (SS), the interosseous sacroiliac ligament (ISL), the anterior sacroiliac ligament (ASL), the posterior sacroiliac ligament (PSL), the long posterior sacroiliac ligament (LPSL) and the pubic symphysis (PS) were modeled. The gluteus medius and maximus were modeled as passive springs as well since muscles also play an important role in joint stabilization (van Wingerden et al., 2004; Vleeming & Schuenke, 2019); **a** view from posteriorly; **b** view from anteriorly

Bone material properties were modelled using isotropic, inhomogeneous bone elasticity (material mapping). Based on the CT intensity, a linear interpolation derived the local density. An exponential approximation of the elastic Young’s modulus with the following equation was used: E = 6.950ρ^1.49^ [18]. This yielded local elastic moduli between 4 and 23 GPa in 40 material classes (Fig. 2). Ligament and passive muscle properties were modelled based on literature values (Table 1, Fig. 3), with stiffness of ligaments from Shi et al. [23], and muscles from Phillips et al. [22]. Attachments were manually placed to best represent consistent landmarks [17]. Ligaments were defined as tension-only and pre-loaded springs. Steinke et al. [24] report sacrotuberous ligament stiffness in cadaver specimens with a mean ± standard deviation of 118 N ± 74 N; a mean of 65 N in females (*N* = 10, median age of 94 years) and a mean of 172 N in males (*N* = 10, median age of 77 years). Existing finite element (FE) studies of the SIJ did not explicitly model ligament pre-tension, but rather ligaments were modelled as tension-only 1D spring elements or 3D truss elements [23, 27, 29]. The pre-tension in this study was generated by shortening the original spring length, with the same pre-tension for TMJ/TFJ = 118 N [24], i.e. shortening to ~ 99.92% of the original ligament length. We deliberately modelled a consistent ligament pre-load for all cases as we wanted to assess the influence of ligament pre-tension on stress and relative joint movement of the SIJ with the covariate of sex-specific joint morphology, but we did not model a sex-specific or even patient-specific model here. This is in scope for future models.

**Table 1:** Stiffness of the ligaments [23] and muscles [22]

Ligament name	Stiffness in N/mm
Anterior sacroiliac ligament (ASL)	700
Interosseus sacroiliac ligament (ISL)	2800
Long posterior sacroiliac ligament (LPSL)	1000
Posterior sacroiliac ligament (PSL)	400
Sacrospinous ligament (SS)	1400
Sacrotuberous ligament (ST)	1500
Pubic symphysis (PS)	(2*500) = 1000
Gluteus maximus	344
Gluteus medius	779

### Contact properties

**Fig. 4:**
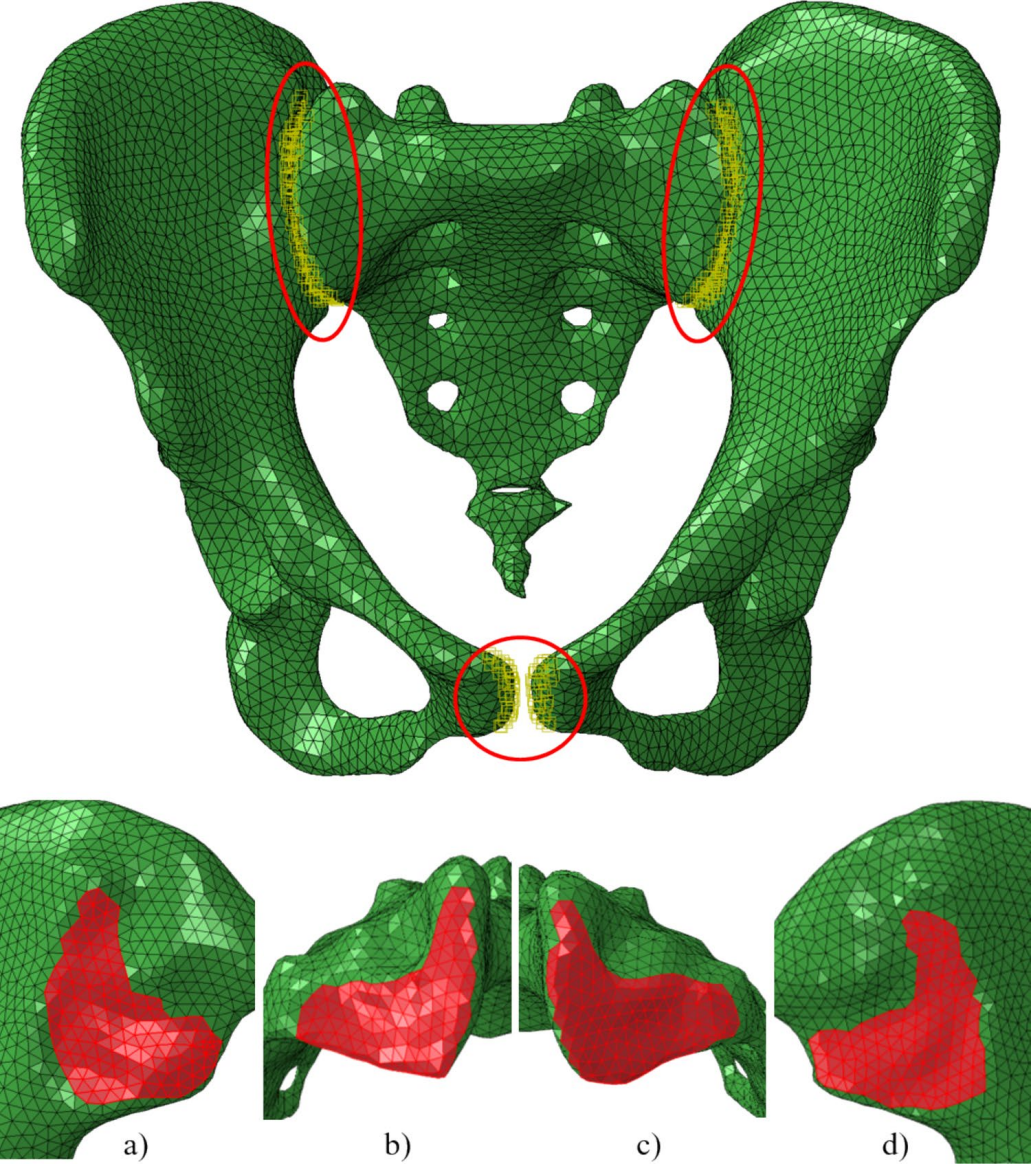
Top: Three contact pairs were identified in the models (specific contact regions were highlighted in red): the left SIJ, the right SIJ and the pubic symphysis. Bottom: Element surfaces representing the SIJ surfaces. **a** Ilium right. **b** Sacrum right. **c** Sacrum left. **d** Ilium left (typical female joint)

Contact was implemented to represent the behaviour of joint cartilage between the ileum and sacrum, but also at the pubic symphysis, with tangential behaviour: friction coefficient of 0.4 [17], and a normal behaviour with non-linear exponential pressure-overclosure contact. The point of zero pressure was set to be the cartilage thickness: 3 mm for the SIJ [16] and 4 mm for the pubic symphysis [15]. The point of zero clearance was set to be the elastic modulus of the cartilage as an approximation (SIJ = 54 MPa, pubic symphysis = 5 MPa [23]). The surfaces with contact definitions are shown in Fig. 4.

### Loads and boundary conditions

The sacrum was held in place at the lumbosacral joint surface. The attachment point for the gluteal muscles was reduced to a single point. Due to the lack of data, this point was chosen to be at the intersection of the implant shaft axis and implant neck axis. This approximation was assumed to be adequate since the area of interest, the SIJ, is relatively far away from the gluteal muscles’ attachment points. The point was mirrored onto the other side as well as the attachment point for the gluteal muscles. Those displacement constraints at the top of the sacrum and muscle attachment points were fixed in space.

**Fig. 5:**
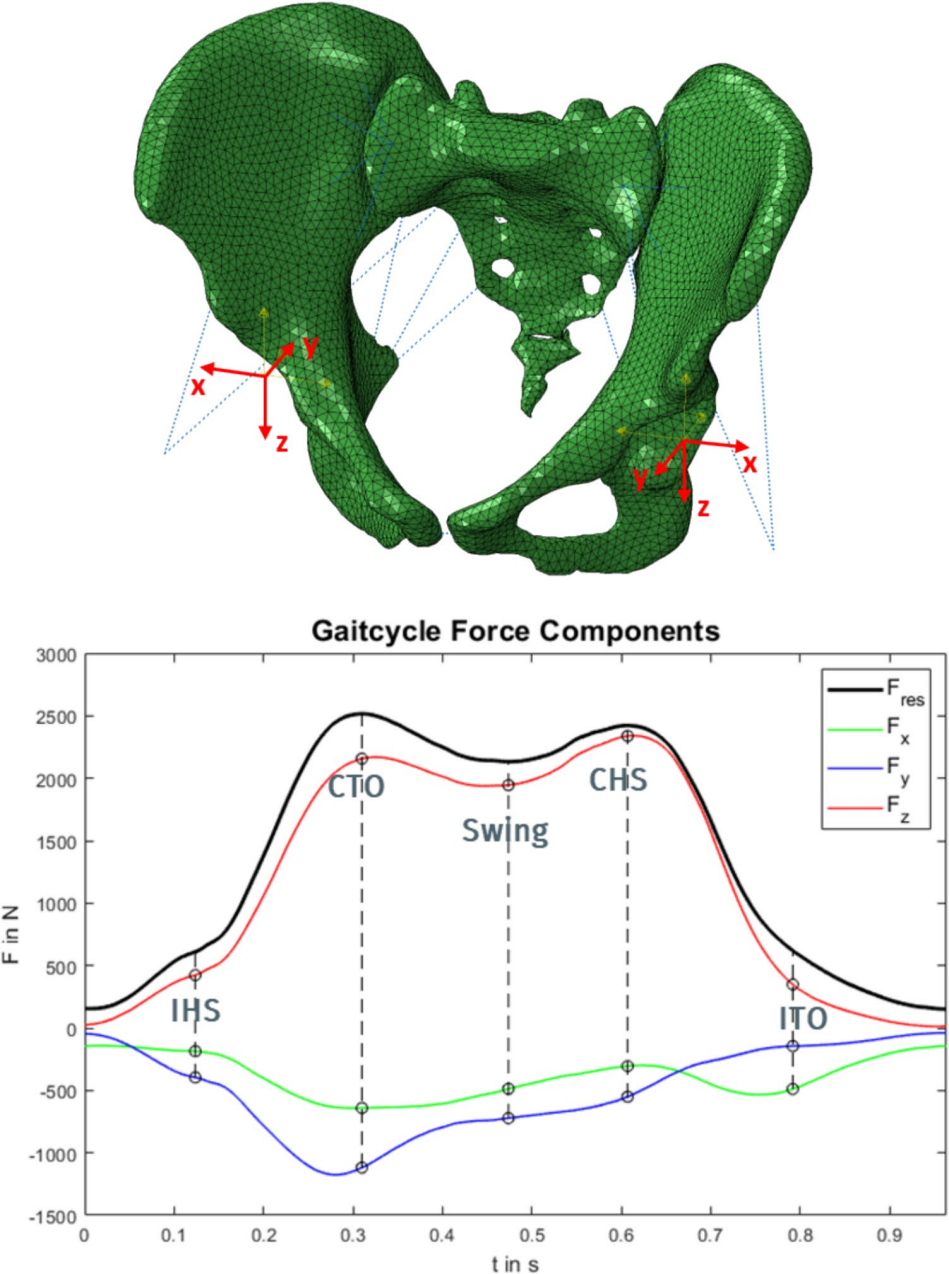
Top: Local coordinate systems in the hip origins (typical female joint). Bottom: Gait cycle force curve with components in the x-, y- and z-directions of the local coordinate system of the left acetabulum. Maximum absolute values are marked with a circle at the specific phases of ipsilateral heel strike (IHS), the contralateral toe off (CTO), the swing phase on the contralateral side, the contralateral heel strike (CHS) and the ipsilateral toe off (ITO)

The loads were taken from our in vivo database. Patient H5L was selected while walking (TrialNr: H5L_050412_1_75). Two loading approaches were chosen (Table 2). Firstly, a generic approach applying the extreme load components that were measured during gait [8] in each direction separately (uniaxial loading x-, y-, or z-direction), either on both sides symmetrically or only one side asymmetrically. The rationale for this lies in the fact that we expected to see the largest effects on stress with the largest loads, but we wanted to separate the load components to see effects of loading directions on joint compression and shear. This results in six different loading scenarios. Additionally, we applied combinations of those extreme loads symmetrically [symmetric xyz] on both sides or asymmetrically [unilateral xyz] only on one side, i.e. 2 additional loading scenarios with the following load levels, Fx = 600 N, Fy = 1200 N, Fz = 2400 N (Fig. 5). Secondly, combined loads during gait were applied in five consecutive gait cycle phases in a quasi-static analysis. The five phases chosen for static simulation were IHS: ipsilateral heel strike, CTO: contralateral toe off, swing phase, CHS: contralateral heel strike and ITO: ipsilateral toe off (Fig. 5).

**Table 2:** Overview of loading approaches and scenarios

Loading approach	Loading components	Symmetry	Axis direction	Load	Short name
Generic loading	Uniaxially	Asymmetrical	Medio-lateral (x-direction in Fig. 4)	Fx = 600 N Unilaterally asymmetrical	Unilateral x
	Uniaxially	Asymmetrical	Antero-posterior (y-direction in Fig. 4)	Fy = 1200 N Unilaterally asymmetrical	Unilateral y
	Uniaxially	Asymmetrical	Cranial-caudal (z-direction in Fig. 4)	Fz = 2400 Unilaterally asymmetrical	Unilateral z
	Uniaxially	Symmetrical	Medio-lateral (x-direction in Fig. 4)	Fx = 600 N Bilaterally symmetrical	Symmetric x
	Uniaxially	Symmetrical	Antero-posterior (y-direction in Fig. 4)	Fy = 1200 N Bilaterally symmetrical	Symmetric y
	Uniaxially	Symmetrical	Cranial-caudal (z-direction in Fig. 4)	Fz = 2400 Bilaterally symmetrical	Symmetric z
	Combined	Asymmetrical	Combined loading	Fx = 600 N, Fy = 1200 N, Fz = 2400 Unilaterally asymmetrical	Unilateral xyz
	Combined	Symmetrical	Combined loading	Fx = 600 N, Fy = 1200 N, Fz = 2400 Bilaterally symmetrical	Symmetric xyz
Gait loads	Combined	Asymmetrical	Combined loading	The specific force values for the whole gait cycle are published and can here downloaded here: Orthoload database case H5L (https://orthoload.com)	Gait loading

### Sensitivity analysis

The sensitivity analysis here assesses the robustness in the joint stability and how much it can be allocated to different sources of uncertainty in the model inputs. Sensitivity was assessed as change in output parameter (von Mises stress in MPa, stress change in %; movement as relative joint translation in mm and rotation in degree, movement change in %) relative to change in input values, i.e. s = Δoutput/Δinput. Input values were ligament reference length (varied 0.998 to 1), ligament stiffness (varied 0.6 to 1.4) and load intensity (patient body weight, varied from 0.6 to 1.4). A negative sensitivity indicated that the output value was decreasing with larger input value, e.g. joint relative translation (as model output) decreases with higher ligament stiffness (as varied model input).

### Mesh convergence study

Two finer grids for the TFJ have been calculated to assess the convergence of the simulation results: a medium (46,873 nodes, 215,058 elements C3D4) and a fine (123,753 nodes, 609,142 elements C3D4) grid. The original grid is called coarse grid (18,162 nodes, 75,837 elements C3D4). The effects of the mesh refinement were evaluated based on the loading scenario symmetric xyz (Table 2), which was the loading scenario with the highest average stresses, strains and relative joint movements.

Differences in relative movement were below 10% in all grids; differences in stress were below 13% in all grids. For calculation, the medium grid took 3.5 × longer duration than the coarse grid, while the fine grid took 11.4 × longer duration than the coarse grid. The coarse mesh was chosen based on the large advantage in computational time savings.

## Results

### Joint loading and relative movement

**Fig. 6:**
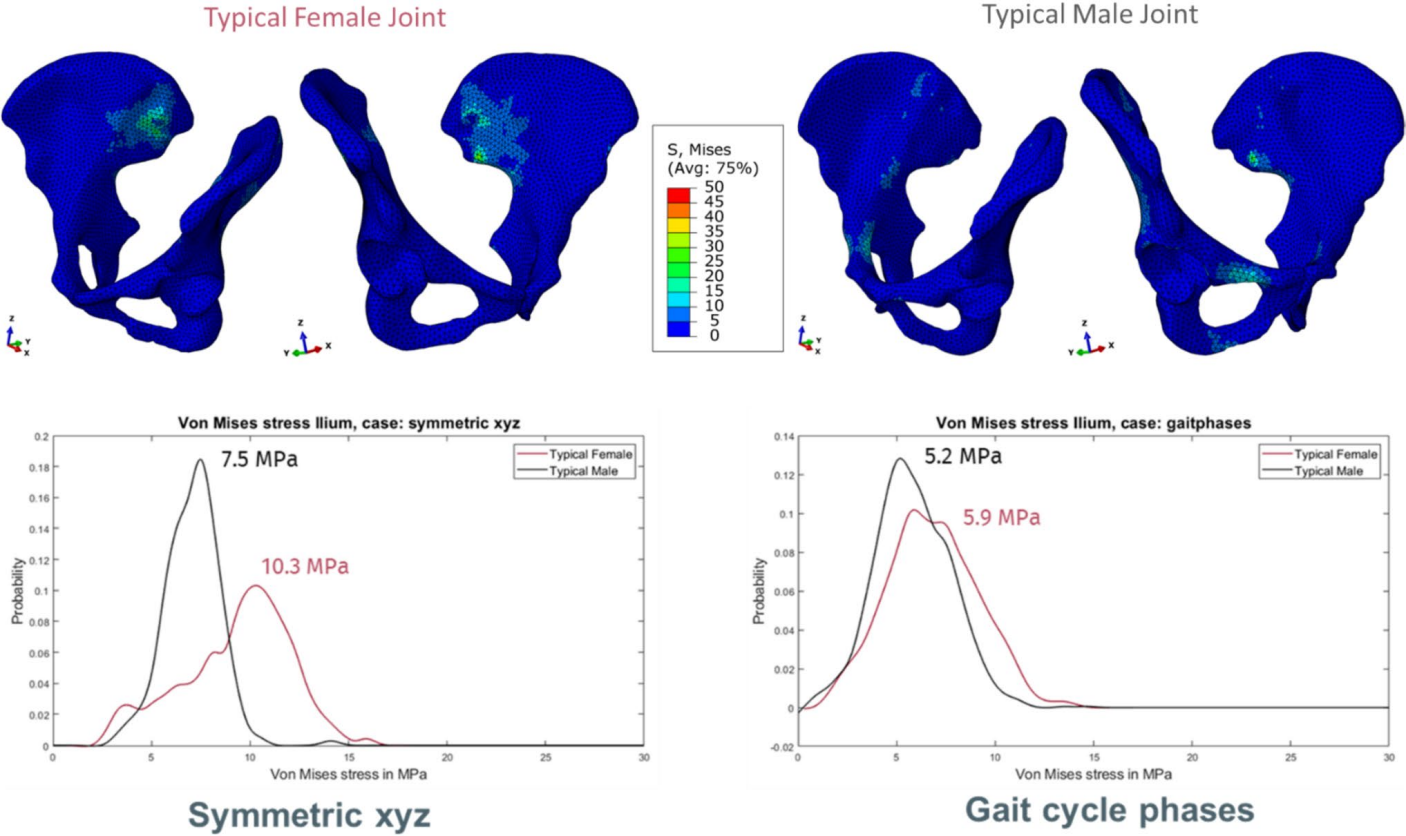
Top: Visualized von Mises stress distribution in the SIJ variants TFJ and TMJ (load scenario symmetric xyz). Legend ranges from 0 up to 50 MPa. At the bottom: von Mises stress distribution for load scenario symmetric xyz (left) and all gait phases (right) for both SIJ variants TFJ and TMJ

In our approach with uniformly scaled bone sizes and loads as well as the same ligament properties, the TFJ generally shows higher stresses compared with the TMJ (Table 3, Fig. 6). In detail, the TFJ was subjected to higher von Mises stresses than the TMJ, but the differences are more pronounced in the generic loading with isolated, uniaxial load components with the most frequent stress for TFJ = 10.3 MPa versus TMJ = 7.5 MPa, while in the more physiological case with combined loading, the most frequent stress was 5.9 MPa for TFJ versus 5.2 MPa for the TMJ.

**Table 3:** Mean von Mises stress values in MPa for generic symmetric load scenarios, asymmetric load scenarios and gait cycle phases

Joint variant	Mean stress in surface …	Symmetric xyz			Symmetric x		Symmetric y			Symmetric z	
Typical female joint	Ilium left	9.7 ± 2.9			5.6 ± 1.4		7.6 ± 4.0			11.0 ± 3.1	
	Ilium right	8.9 ± 2.5			5.8 ± 1.7		8.8 ± 4.1			9.5 ± 2.6	
Typical male joint	Ilium left	6.8 ± 1.3			4.2 ± 1.2		8.8 ± 3.9			8.0 ± 1.5	
	Ilium right	7.4 ± 1.5			4.4 ± 1.4		8.7 ± 3.9			8.8 ± 2.1	
Joint variant	Mean stress in surface …	Asymmetric xyz			Asymmetric x		Asymmetric y			Asymmetric z	
Typical female joint	Ilium left	8.1 ± 2.2			8.2 ± 2.0		6.7 ± 2.7			9.7 ± 2.5	
	Ilium right	4.9 ± 1.4			1.8 ± 0.7		5.7 ± 1.5			5.0 ± 1.5	
Typical male joint	Ilium left	6.8 ± 1.5			5.7 ± 1.6		6.8 ± 2.7			8.3 ± 1.5	
	Ilium right	4.2 ± 1.3			2.8 ± 0.7		5.5 ± 1.4			4.8 ± 1.3	
		Gait cycle phase									
Joint variant	Mean stress in surface …	IHS		CTO		Swing			CHS		ITO
Typical joint female	Ilium left	5.9		7.0		7.8			9.1		7.7
	Ilium right	8.3		7.1		4.6			4.7		6.2
Typical joint male	Ilium left	3.9		5.3		6.7			7.8		5.5
	Ilium right	8.0		6.3		4.1			4.7		6.0
Averaged 99th percentile, mean and median von Mises stress values across all generic load scenarios and gait cycle phases. Values in MPa											
	von Mises stress (all loads)										
Joint variant	99 th percentile		mean					median			
Typical female joint	11.6 ± 3.7		7.1 ± 2.0					7.4 ± 2.2			
Typical male joint	9.5 ± 3.2		6.2 ± 1.7					6.3 ± 1.7

Relative joint translations and rotations are small in the SIJs (Table 4). The TFJ appears to have larger translations than the TMJ (Table 4) in certain loading regimes.

**Table 4:** Relative translations and rotations during the generic symmetric load scenarios, asymmetric load scenarios and gait cycle phases

Relative translation (mm)		Side	Symmetric xyz		Symmetric x	Symmetric y	Symmetric z			Asymmetric xyz			Asymmetric x	Asymmetric y	Asymmetric z
Typical female		Left	0.6		0.2	4.5	0.6			0.6			0.7	2.1	0.6
		Right	0.7		0.2	4.8	0.9			0.1			0.3	1.8	0.0
Typical male		Left	0.3		0.1	4.8	0.6			0.3			0.2	2.5	0.4
		Right	0.3		0.1	5.7	0.4			0.0			0.3	2.5	0.1
Relative translation (mm)		Side	IHS		CTO	Swing	CHS			ITO					
Typical female		Left	0.0		0.6	0.6	0.7			0.2					
		Right	0.7		0.2	0.1	0.1			0.7					
Typical male		Left	0.2		0.2	0.3	0.4			0.2					
		Right	0.3		0.2	0.1	0.1			0.3					
Relative rotation (°)		Side	Symmetric xyz		Symmetric x	Symmetric y		Symmetric z		Asymmetric xyz		Asymmetric x		Asymmetric y	Asymmetric z
Typical female		Left	0.9		0.9	7.2		1.8		0.7		2.7		4.5	1.4
		Right	0.8		1.0	7.6		1.6		0.5		1.4		2.8	0.3
Typical male		Left	0.6		0.2	7.6		1.3		0.5		1.1		5.8	1.5
		Right	0.6		0.3	7.2		1.3		0.2		1.0		3.5	0.7
Relative rotation (°)	Side			IHS	CTO	Swing			CHS		ITO				
Typical female	Left			0.4	0.9	0.7			1.0		1.3				
	Right			0.9	1.0	0.4			0.3		1.0				
Typical male	Left			0.8	0.7	0.6			1.0		0.5				
	Right			1.2	0.7	0.2			0.6		0.4				
Translations and rotations in all SIJ variants averaged over all load scenarios, generic scenarios and gait cycle phases															
**SIJ variant**	**Mean translations in mm**					**Mean rotations in °**									
Typical female	0.9 ± 1.2 mm					1.7 ± 1.9°									
Typical male	0.8 ± 1.4 mm					1.5 ± 2.0°									

### Model analysis of joint stability (sensitivity analysis)

Sensitivity of the model results is inconsistent (Table 5): Higher ligament reference length, which means a lower pre-tension, leads to higher stresses in the TFJ (2.11 mean sensitivity), but also at the same time lower stresses in the TMJ (− 8.97 mean sensitivity). Higher ligament stiffness leads to slightly higher stresses (mean sensitivity TFJ = 0.17, TMJ = 0.13). Also, and more profoundly, higher load intensity (higher patient body weight) leads to higher stresses (mean sensitivity TFJ = 0.54, TMJ = 0.58).

**Table 5:** Mean sensitivity values for TFJ and TMJ for the input parameters ligament reference length (reduced pre-tension), ligament stiffness and load intensity (patient body weight) to the output values of mean von Mises stress in the ileum, mean joint translation and mean joint rotation

Parameter name	Parameter variation	TFJ Mean sensitivity stresses	TFJ Mean sensitivity translation	TFJ Mean sensitivity rotation	TMJ Mean sensitivity stresses	TMJ Mean sensitivity translation	TMJ Mean sensitivity rotation
Ligament reference length	0.998 to 1	2.11	71.04	43.09	− 8.97	33.64	4.02
Ligament stiffness	0.6 to 1.4	0.17	− 1.14	− 0.90	0.13	− 1.06	− 0.89
Load intensity	0.6 to 1.4	0.54	1.09	0.91	0.58	1.10	0.88

Higher ligament reference length, or equivalently lower pre-tension, leads to more joint movement consistently, but with a much larger sensitivity for the TFJ with 71 for translation and 43 for rotation, and for the TMJ, 34 for translation and 4 for rotation. Mean sensitivity of movement to ligament stiffness is around − 1.1, while mean sensitivity of movement to load intensity (higher patient body weight) is about + 1.1.

We assessed the position of muscle attachment points, and the influence of 10 mm medial or 20 mm lateral shift on stress or movement values was below 3%. The effect of SIJ contact type (exponential vs. linear contact) was high with stresses with linear contact type: 37% higher for TFJ, 49% higher for TMJ; translations: 22% lower for TFJ, 39% lower for TMJ; rotations: 2% lower for TFJ, 22% lower for TMJ. The effect of element type (linear vs. quadratic) was also pronounced with quadratic elements, stresses: 23% higher in TFJ, 52% higher in TMJ, translations: 2% lower in TFJ, 14% lower in TMJ, rotations: 3% higher in TFJ, 8% lower in TMJ. Other sensitive modeling parameters were the contact friction (0.2 to 0.6), which was sensitive to translation (− 0.83 in TFJ, − 0.48 in TMJ), and contact overclosure (surrogate of cartilage thickness), which was sensitive to stress (0.65 in TFJ, 0.91 in TMJ) and translations (− 0.52 in TFJ, − 1.06 in TMJ).

## Discussion

The objective of this study was to investigate the effect of ligament pre-tension on joint surface stress and relative movement using FE models of the SIJs. We hypothesized that the influence of ligament pre-tension on stress and relative joint movement of the SIJ is morphologically sex-specific and larger than that of load intensity in terms of body weight.

Our results indicate that there is almost a linear behaviour of joint movement (translation and rotation) with patient weight and ligament stiffness, and a non-linear effect on stress changes of those parameters. However, movement is also very sensitive to ligament reference length (or pre-tension) compared to stress values. Relative joint translation/rotation is by far most influenced by the pre-tension level of the ligaments, and much more than by the patient’s body weight (load intensity), which exhibits similar sensitivity as ligament stiffness to relative joint motion, confirming our hypothesis for joint movement. Concerning local stress, the mean joint stress is also much less sensitive to load intensity than ligament pre-tension, also confirming our hypothesis. Interestingly, the mean joint stress can decrease with *lower* pre-tension in TMJ, but also with *higher* pre-tension in TFJ. Thus, ligament pre-tension should always be considered in relation to other factors such as joint morphology and attachment points, but also ligament stiffness. Ligament pre-tension may be one aspect contributing to sex differences [19]. Overall, our results confirm those of previous studies, which have shown that decreasing ligament stiffness causes increasing SIJ motion [9].

Considering the fact that up to 30% of LBP are caused by the SIJs, a better understanding of factors influencing SIJ movement and loading is essential for the treatment of this challenging patient cohort. While ligament stiffness has previously been described as a potential cause of SIJ pain, the exact mechanism of pain development has yet to be understood [5]. In this regard, our results provide insight into the complex interplay between ligament tension and joint movement and loading. Interestingly, the ligament pre-tension’s effect on SIJ loading is different in males and females, which may be one factor to be considered in the sex-specific differences of LBP incidence. This is especially of note as in women ligamentous laxity is a common effect of hormone changes during pregnancy and thus occurs more frequently than in men [5]. As we show that in women lower pre-tension of SIJ ligaments leads to higher stresses, this may be an important factor in female LBP development. Future studies also need to investigate how higher joint movement caused by lower ligament stiffness affects pain development as we show that in both males and females, lower ligament pre-tension leads to a higher joint movement. Previous studies have not found a significant effect of joint movement on pain but were limited to low patient numbers [25].

Our investigation required a number of assumptions and had numerous limitations: we tried to use consistent scaling and loading by transforming the individual geometries to a common reference model. However, we still used two different morphologies and were able to show that morphology seems to have effects on the joint stresses and even more so on the joint movement. By uniformly scaling the geometries to a common reference and applying the same load profile, we aimed to isolate the effect of joint morphology on the sensitivity to ligament pre-tension. While absolute stress and movement values might differ in unscaled, individually loaded models, the observed *relative* sensitivities and the comparison between male and female typical joints under identical loading conditions provide valuable insights into the underlying biomechanical principles. We did not model individual ligament stiffness, and attachments were placed manually. Methods based on detailed anatomical atlases or statistical shape models could further enhance accuracy of landmark placement especially in studies focusing on variability of attachments. We did not utilize a musculoskeletal model to derive individual active muscle forces, but only used the measured hip joint reaction forces of one female subject. Future studies incorporating musculoskeletal models could provide a more comprehensive understanding of the combined effects of active and passive structures. However, with different approaches of load application, we showed that the stress and joint movement results are sensitive to the boundary conditions [14] such as the loading model as well. Especially uniaxial, isolated loading (anterior–posterior direction) seems to lead to much higher movement and also higher stress values. Future work will indeed focus on a more in-depth investigation of various physiological (realistic) loading scenarios, potentially including simulations of specific activities of daily living and different gait patterns, to further refine our understanding of SIJ biomechanics. Also, integrating finite element modeling with artificial intelligence holds promise for future SIJ biomechanics research by accelerating computations, improving clinical applicability and enabling personalized models that capture individual patient variability [12, 30].

Potential therapy targets for excessive stresses in the SIJ or hypermobility could be confirmed such as avoiding isolated, uniaxial loading, weight loss, but also the underappreciated ligament pre-tension/ligament stiffness, which might be even more promising. Our findings suggest distinct clinical implications based on the sex-specific biomechanical response of the SIJ to ligament pre-tension. In typical female SIJs, particularly during physiological states potentially associated with increased joint laxity (e.g. pregnancy due to hormonal influences), our simulations indicate heightened vulnerability to stress and relative movement under isolated anterior–posterior loading. This highlights the importance of patient education and activity modification to minimize movements in this direction. Furthermore, therapeutic interventions, ranging from targeted physiotherapy aimed at stabilizing the joint to potentially considering surgical fusion in recalcitrant cases, could focus on limiting excessive anterior–posterior motion. Conversely, in typical male SIJs, our model suggests that higher ligament pre-tension might contribute to restricted joint motion and elevated stress levels. This could imply that therapeutic strategies for some male patients might explore interventions aimed at modulating ligament tension or addressing factors contributing to this increased pre-load, although further research is needed to identify specific targets and techniques. Overall, our in silicon findings underscore the potential for sex-specific considerations in the diagnosis and management of SIJ-related pain, warranting future clinical investigations to validate these biomechanical insights and explore the efficacy of targeted therapeutic approaches.

## Conclusions

In conclusion, this in silicon study provides novel insights into the biomechanical role of ligament pre-tension in the sacroiliac joint, revealing distinct morphologically sex-specific responses to variations in this parameter and load intensity. Our findings demonstrate that ligament pre-tension significantly influences both joint stress and relative movement, with a more pronounced effect observed in the typical female SIJ, particularly regarding joint motion. Notably, the sensitivity analysis suggests that, for joint movement, the effect of ligament pre-tension is more substantial than that of body weight. Furthermore, our simulations indicate that isolated anterior–posterior loading may pose a higher risk to joint stability, especially in female morphologies. These biomechanical distinctions between typical male and female SIJs under varying ligament pre-tension suggest potential sex-specific considerations for understanding SIJ pain development and for guiding future research into targeted diagnostic and therapeutic strategies. Ultimately, in vivo validation of ligament pre-tension and its influence on SIJ biomechanics is crucial for translating these computational findings into clinical practice in the future. All in all, assessments of pre-tension of passive structures need more attention and should be investigated in situ. An injury that changes the material characteristics of any ligament will influence the structural behaviour, local stresses and most likely the kinematics of the whole articulating structure.
